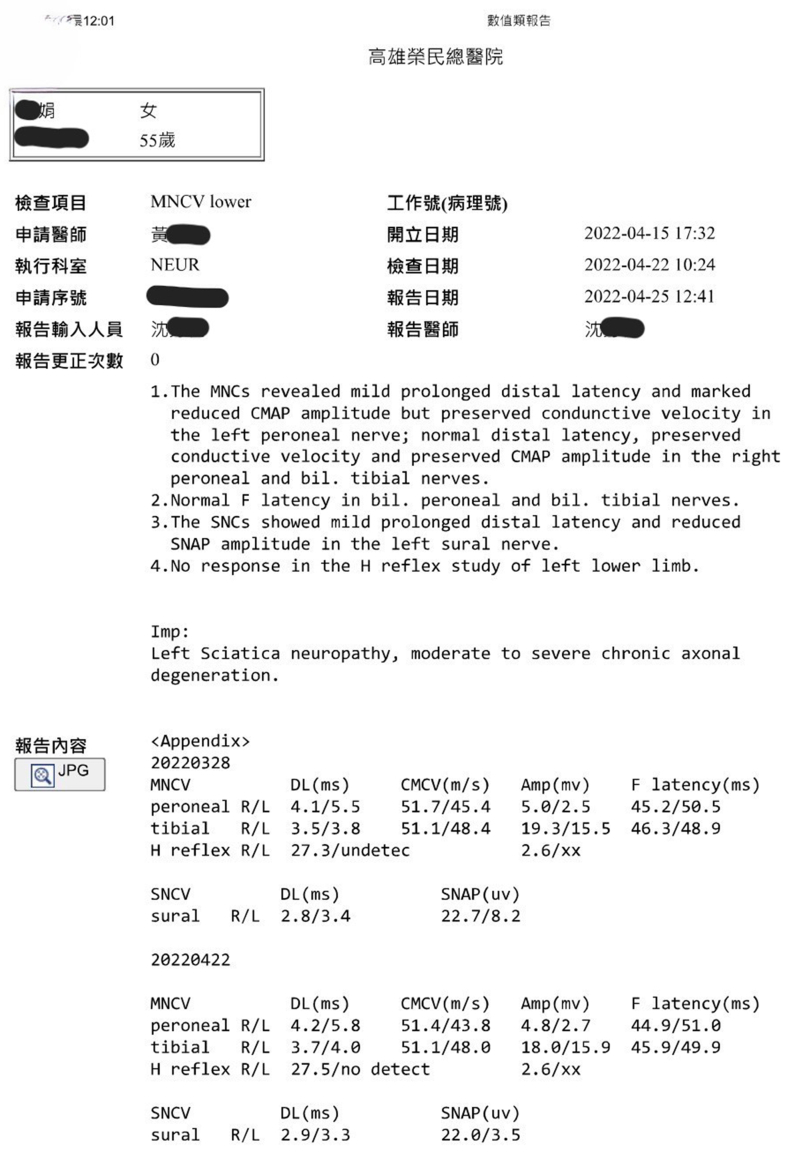# Feasibility of a promising pulsed electrostimulator for rapid motor recovery of foot drop

**DOI:** 10.1016/j.heliyon.2024.e25176

**Published:** 2024-02-01

**Authors:** Yu-Cheng Chang, Yuan-Ping Chao, Shin-Tsu Chang

**Affiliations:** a Department of Medical Research and Education, Kaohsiung Veterans General Hospital, Kaohsiung, Taiwan; b Department of Clinical Education, Dalin Tzu Chi Hospital, Chiayi, Taiwan; c Department of Clinical Education, Hualien Tzu Chi Hospital, Hualien, Taiwan; d School of Medicine, Hualien Tzu Chi University, Hualien, Taiwan; e Department of Ophthalmology, Tri-Service General Hospital, Taipei, Taiwan; f Department of Physical Medicine and Rehabilitation, Kaohsiung Veterans General Hospital, Kaohsiung, Taiwan; g Department of Physical Medicine and Rehabilitation, Tri-Service General Hospital, School of Medicine, National Defense Medical Center, Taipei, Taiwan

**Keywords:** Foot drop, Transcutaneous electrical nervous stimulation, Pulsed radiofrequency

## Abstract

**Purpose:**

Foot drop still occurs in clinical practice, including in our case. Treatments for foot drop vary based on its etiology and severity of symptoms. Hence, in intractable foot drop cases, an invasive surgical intervention is needed. Here, we introduce a special noninvasive technique to treat our patient's foot drop. In this approach, we applied STIMPOD NMS460 neuromuscular stimulator device (STIMPOD NMS460), which is a low-frequency (10 Hz or less) transcutaneous electrical nerve stimulation (TENS) device with a pulsed radiofrequency (PRF) component. We are eager to know how effective the device is in treating foot drop, and we compared it with two kinds of surgical interventions.

**Materials and methods:**

The device settings are 5 Hz in frequency and 30mA in current amplitude. The device was applied on her left side at the L4 and L5 regions and at the fibular head. Each therapy session consists of individual 15-min treatments on these two body areas, and it only takes a total of 30 minutes. We recorded the change in ankle dorsiflexion degrees and muscle strength of our patient.

**Results and Conclusions:**

To our surprise, our patient's actual treatment status through STIMPOD NMS460 showed more effective recovery and no specific side effects than surgical interventions in similar conditions. Besides, after a three-month intervention, her affected ankle dorsiflexion recovered to almost her usual status. The reason why this device has such an effect may be that it has the benefits of TENS and PRF. Besides, some studies have revealed the nerve-repair effect of TENS and PRF. In conclusion, we believe that this device is fairly promising and may be qualified to be used in other patients with foot drop.

## 1 Introduction

Foot drop is a longstanding issue that can be caused by a variety of diseases or injuries. According to the literature [1], the most common etiology of foot drop is peroneal neuropathy, often a compressive neuropathy of the lower extremities from causes such as positioning during surgery, compression stockings, fibula fractures, etc. [2]. Among these causes, positioning during surgery is an uncommon etiology of iatrogenic injury in current society. In our case, there is a high likelihood that her placement in a lithotomy position during surgery was the main reason for our patient's foot drop. In a previous study, researchers reported that each hour in a lithotomy position increases the risk of motor neuropathy nearly one hundred-fold [3]. As a result, in the following content, we will focus on the foot drop caused by compressive neuropathy. As expected, there is a need for an optimized strategy for foot drop problems.

Treatments of compressive neuropathy vary based on the etiology and severity of symptoms. The most common and effective nonsurgical treatment options are physical therapy with nerve gliding and nerve flossing [4]. Padding of the fibular head is another option that can work at night to prevent compression while sleeping [5]. Orthotic intervention is an additional option, involving items such as a lateral wedge shoe and ankle foot orthosis [6]. An alternative treatment to be considered before surgical intervention is hydrodissection [7]. Hydrodissection involves injecting a nonirritating solution (e.g., saline, anesthetics, steroids) around the nerve to reduce the pressure from surrounding structures. When compressive neuropathy is refractory to nonsurgical treatments, surgical decompression or nerve transfer is the mainstay treatment option [8,9]. Notably, Andrea et al. stated that if the symptoms of peripheral nerve entrapment persist for 2–3 months, permanent nerve damage will ensure [10]. Hence, surgical intervention should be adopted in this situation. However, a longer recovery time or side effects might occur under noninvasive treatment or invasive treatments.

Here, we introduce a special noninvasive technique to treat our patient's foot drop. In this approach, we applied STIMPOD NMS460 neuromuscular stimulator device (STIMPOD NMS460), which is a low-frequency (10 Hz or less) transcutaneous electrical nerve stimulation (TENS) device with a pulsed radiofrequency (PRF) component. It has similar advantages of a normal TENS device, with the added benefits of PRF, all in one waveform delivered to the patient noninvasively. In this case report, we discuss our patient with foot drop from compressive neuropathy, likely due to lithotomy positioning during surgery, who quickly recovered from her illness with this device as well as why STIMPOD NMS460 had such an effect.

### 1.1 Case presentation

A 55-year-old female hair dresser developed a pimple on her right vulva last March. She presented to a local hospital for treatment, where she was told that the lesion was just acne. However, after 2 years, it became larger and more uncomfortable, so she returned to the hospital for a biopsy of the lesion. Unfortunately, the pathology showed basal cell carcinoma. The doctor recommended she receive MRI for a more detailed examination. However, the scheduling would have taken one month. The patient felt very uneasy regarding such a long period, so she came to the Dermatology out-patient department in another local hospital for help on 2022/3/4 through her friend's recommendation. She was admitted on 2022/3/7 and transferred to the Plastic Surgery ward for operation of surgical resection. She underwent surgery on 2022/3/10, which started at 09:08 a.m. and ended at 10:50 a.m. The total surgical time was 1 hour and 42 minutes. After the surgery, both feet were numb in the recovery room. When she returned to the ward, her right foot had sensation and motor function, but her left foot from the calf down had lost sensation and mobility. Prior to the surgery, she had often exercised at the gym, danced, and loved outdoor activities. At that time, the doctor said it was likely the anesthesia had not worn off yet. However, the next day, her left foot was still numb, with a sensation described “as if there was a thick layer of skin on the outside.” Additionally, her left foot was still immobile, which made her unable to stand or perform her daily activities of living including going to the restroom or putting on her shoes. Her symptoms continued to cause anxiety, so she followed up with her physicians. The anesthesiologist said it was probably because of the lithotomy position, and the surgeon said it was probably because the nerves were affected by the sedation used.

**Fig. 1:**
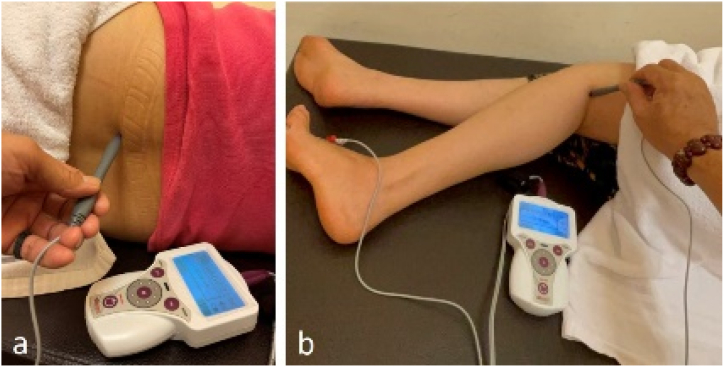
The location for application of STIMPOD NMS460. The device was applied on her left side at the L4 and L5 regions (1a) and at the fibular head (1b). Each session took only 30 minutes.

Later, the patient was fearful of her future and searched the internet for answers, learning that her problem was named foot drop. Hence, she went to our department of rehabilitation for help. Under an electromyography examination (Supplementary file 1), it showed she injured her left peroneal nerve and left sural nerve. Combined with history taking and the literature review [1,2], we made a diagnosis of left foot drop with injured left peroneal nerve and left sural nerve, likely caused by lithotomy position when anesthesia. After discussing the treatment options with this patient, we decided to use a special device, STIMPOD NMS460, to treat her foot drop; besides, we obtained the patient's consent to record her treatment process and write it up as a case report. Her treatment began on 2022/5/12. The device was applied on her left side at the L4 and L5 regions and at the fibular head (Fig. 1a and 1b). Each therapy session consists of individual 15-min treatments on these two body areas, and it only takes a total of 30 minutes. After the first and second session, her left toes' motor function progressed from minimal movement to upward extension; however, the dorsum of the foot could still not dorsiflex upward. In addition, the “thick skin” sensation of her left foot had improved. Unfortunately, her third session was suspended because the patient contracted COVID-19. During the days of isolation at home, she diligently performed rehabilitation exercises and massages at home, including flexion and extension of her lower left leg. After she recovered from COVID-19, she resumed STIMPOD NMS460 therapy on 2022/6/15 and received the therapy three times a week.

**Fig. 2:**
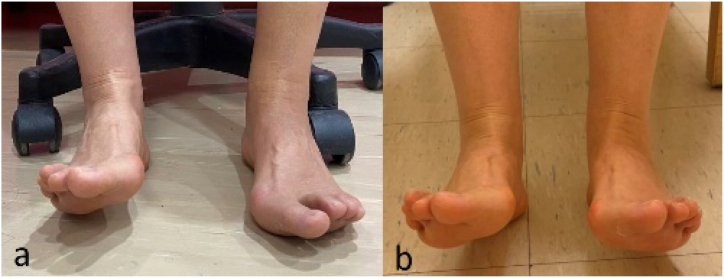
Two photos show her treatment effects. In the third session, her left dorsiflexion (affected side) was 0–5° active range of motion (AROM) and a meanwhile her right dorsiflexion (healthy side) was 0–30° AROM (2a). After a three-month intervention, her left dorsiflexion was 0–27° AROM, which was almost the same as her healthy foot (2b).

**Fig. 3:**
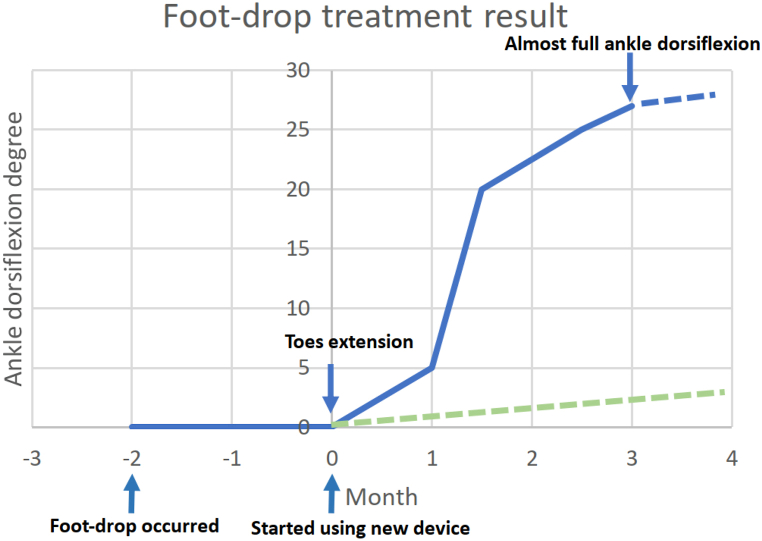
Improvement of dorsiflexion of the case in various intervals of recovery. Solid Line shows progress began immediately in the first month, with rapid improvement within the second month. After a total of 3 months, the patient's dorsiflexion degree almost reached the same degree as the normal side. On the other hand, dotted line shows possible recovery duration without any intervention.

In the third session, her left dorsiflexion (affected side) was 0–5° active range of motion (AROM). In comparison, her right dorsiflexion (healthy side) was 0–30° AROM (Fig. 2a). After a three-month intervention, her left dorsiflexion was 0–27° AROM, which was almost the same as her unaffected foot (Fig. 2b). The patient felt very satisfied with such results. We recorded our patient's progress in the degree of dorsiflexion (Table 1 and Fig. 3). She had slow progress in the first month with significant advancement during the second month. After a total of 3 months, the patient's dorsiflexion degree almost reached the same degree as the normal side (Table 1).

**Table 1:** The progression of patient's dorsiflexion degree. AROM is active range of motion, and PROM is passive range of motion. Clinically, PROM is more objective to prevent patients from not wanting to move for some reasons, but excluding such conditions. On the other hand, AROM is the angle that patients can actually move, and our patient's AROM was almost the same as that of the healthy side after three-month treatment, which shows that the treatment effect is promising and satisfies our patient.

Time for follow-up after intervention	Left dorsiflexion (affected side)		Right dorsiflexion (healthy side)	
	PROM (degree)	AROM (degree)	PROM (degree)	AROM (degree)
0 month	0–5	0	0–35	0–30
1 month	0–10	0–5	0–35	0–30
1.5 months	0–35	0–20	0–35	0–30
2.5 months	0–35	0–25	0–35	0–30
3 months	0–35	0–27	0–35	0–30

## 2 Discussion

Our case involved a middle-aged female who had a significant foot drop problem. However, under this special treatment, STIMPOD NMS460, she obtained a very quick motor recovery of her ankle dorsiflexion.

Despite the short time of treatment, she recovered to near her prior baseline status. Moreover, no side effects including scar formation, painful neuroma formation, or any discomfort occurred in our case or were reported by other users. STIMPOD NMS460 has been successfully used for several medical conditions that have been shared on the device website [11]. To the best of our knowledge, only a complex regional pain syndrome case has been published by Phyllis, and this device has not been used in patients with foot drop [12]. Nevertheless, Phyllis's study existed some disadvantages. First, they used four therapeutic approaches to treat the patient, so it could not be fully attributed to the efficacy of STIMPOD NMS460. Second, they used a rough way, how to hold the teddy bear, to record recovery of mobility instead of degree. Hence, we are pleased to present the first case report of foot drop treatment outcomes with STIMPOD NMS460 and record our patient's recovery of degree of her dorsiflexion.

Some previous studies have reported that poor prognostic factors for nerve regeneration and functional recovery include advanced age, male sex, increased time to intervention, and axonal injury [[13], [14], [15], [16]]. We compared our case to the outcomes of 5 patients previously reported who underwent surgical repair, nerve transfer or decompression surgery, of their foot drop (Table 2). We included the following factors: age, sex, time to intervention, and whether there was axonal loss (Table 2). First, comparing the nerve transfer cases with our case, the two patients were much younger than our patient [9]. Although one of the cases had a much longer time to intervention, it took these two patients 25-fold and 15-fold of the time, respectively, to recover their dorsiflexion strength from 0 to 4, the same recovery extent as that in our patient. Nerve transfer is an invasive intervention, with high risk of leaving scars or painful neuroma formation, often reducing patients' quality of life. Second, comparing decompression surgery cases with our case, two of their patients did not have axonal injury, and had a shorter time to intervention, and had better pre-intervention muscle strength [17]. However, after decompression surgery, it took the patients the same period of time as our case to recover only 2 grades of dorsiflexion strength. Similar to nerve transfer, decompression surgery is also an invasive treatment that may cause scar formation or even complex regional pain syndrome, which was reported in their study [17]. These results demonstrate a similar benefit from a noninvasive device with much lower risk and an absence of side effects. Fig. 3 outlines the progression of our patient's improvement over time. She showed mild recovery within the first month, with the most rapid results seen during the second month. After a total of 3 months, the patient's dorsiflexion degree of her affected side almost reached the same degree as her normal side.

**Table 2:** The comparison of our case and the other invasive treatments.

Study	Total Case numbers	Age	Sex	Nerve injury type	Time to intervention (months)	Preintervention dorsiflexion strength (/5)	Intervention	Time for follow-up checking after intervention (months)	Postintervention dorsiflexion strength (/5)	Comment
Our case	1	55	F	Axonal injury	2	0	STIMPOD NMS460	1	4	
Rahul KN et al. [9]	14	25	F	Axonal injury	2	0	Nerve transfer	25	4	Younger age, but prolonged recovery time
		15	F	Axonal injury	6	0	Nerve transfer	15	4	Younger age, longer time to intervention, but prolonged recovery time
Bilal T et al. [17]	15	54	F	Demyelinating injury without axonal injury	0.5	2	Decompression surgery	1	4	Shorter time to intervention, better preintervention muscle strength, but only 2 grades recovery in the same time
		55	F	Demyelinating injury without axonal injury	0.5	1	Decompression surgery	1	3	Shorter time to intervention, better preintervention muscle strength, but only 2 grades recovery in the same time

**Fig. 4:**
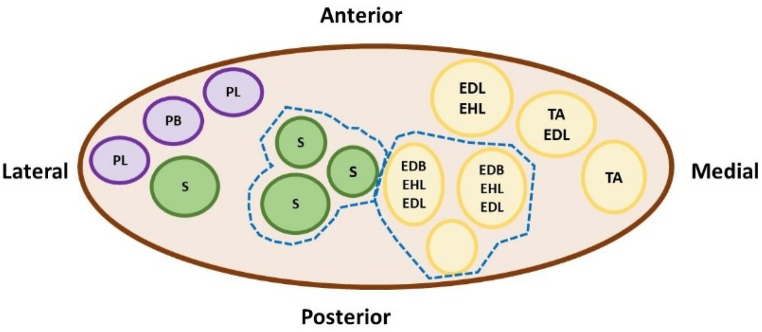
Schematic explanation of nerve anatomical location in the lower leg. Dotted borders: Ankle and toe dorsiflexors; Solid borders: cutaneous sensory fibers; Absent borders: ankle evertors. EDB: Extensor digitorum brevis; EDL: extensor digitorum longus; EHL: extensor hallucis longus; PB: peroneus brevis; PL: peroneus longus; S: sensory fibers; TA: tibialis anterior. The common peroneal nerve wraps around the fibular neck with the medial nerve fascicles innervating the tibialis anterior. The lateral nerve fascicles innervate the extensor digital longus and extensor halluces longus. Hence, it is proposed that compression at the fibular head will cause more damage to ankle dorsiflexion muscles than to toe extensor muscles, which may explain this patient's recovery pattern.

The progression of our patient's motor recovery from distal toe extension to ankle dorsiflexion can be explained by the nerve anatomy in the region. Based on Fig. 4, the anatomy of the common peroneal nerve around the fibular neck shows that the nerve fascicles that control the tibialis anterior are the most medial. On the other hand, the nerve fascicles that control the extensor digital longus and extensor hallucis longus are located laterally. Thus, we proposed that compression at the fibular head will cause more damage to ankle dorsiflexion muscles than to toe extensor muscles, which may be the reason why this patient recovered her toe movement first. To the best of our knowledge, the recovery of foot drop starting from the distal aspect under this device is the first report in the field of peripheral neuropathy. However, there is a previous study reporting that muscle power usually returns most rapidly in large muscles and slowest in small muscles used for fine movements, which is contradictory to our inference [18].

Among their users, one female patient who suffered from Bell's palsy, which similar to our case involves peripheral motor neuropathy, markedly improved after receiving STIMPOD NMS460 treatment [19]. At the beginning, most of the Oxford muscle grading scale of her palsied facial muscles was zero (0/5). After treatment with this device for 1.5 months, most of her palsied facial muscles scored up to 3/5, and some muscles scored 3+/5 and 4-/5.

Additionally, STIMPOD NMS460 has been successfully used in several medical conditions that have been shared on the device website, including complex regional pain syndrome, polyarthritis, tennis elbow, golfer's elbow, sciatic pain, foot pain, and knee pain [11]. For example, the polyarthritis case was a 67-year-old male patient, who suffered from an inability to close his hands fully for over 20 years. This problem made him give up his beloved guitar. Fortunately, he was able to start STIMPOD NMS460 therapy, needing only two and a half minutes each session on the front and the back of his wrist for effects to be seen. A few days later, he was able to return to his baseline, close his hands completely, and continue playing guitar again [11].

STIMPOD NMS460 possesses the treatment effects of low-frequency TENS and PRF. In addition, this device also integrates a nerve-locating technology named the “nerve mapping probe”, which enables operators to locate nerves and evaluate the improvement in damaged nerves after treatment. The effect of low-frequency TENS is typically explained by the release of endogenous opioids to achieve pain relief [20]. Another study compared the effects of low-frequency TENS (4 Hz) and those of high-frequency TENS (100 Hz) on nerve lesions and showed that high-frequency TENS and nontreated nerves had a similar profile with widespread signs of degeneration. In contrast, low-frequency TENS led to increased nerve regeneration, presenting histological features like those of control nerves [21]. These findings implied that applying high-frequency TENS may lead to negative impacts on injured nerves, while low-frequency TENS may promote nerve regeneration. For PRF, its waveform deep to the affected area transcutaneously creates electromagnetic effects. Some studies have reported that the presence of an electromagnetic field prompts the up-regulation of gene expression, including cartilage repair, neuronal differentiation, and skeletal muscle cell growth and differentiation [[22], [23], [24]]. These findings indicate that cell proliferation and tissue compensation can be manipulated, suggesting that electromagnetic fields may become a key regenerative treatment for these tissues. Additionally, a study revealed that PRF can promote neurological repair and improve neuropathic pain by activating the expression of glial cell line-derived neurotrophic factor (GDNF), which suppresses the expression of glial ﬁbrillary acidic protein (GFAP) [25]. GFAP is a marker of astrocytes in the central nervous system, which is related to the triggering of the sense of pain [26]. GDNF is relevant to regulating the transmission of pain signals, especially in neuropathic pain, and is found to be able to reverse the changes induced by neural injury [27,28]. Unfortunately, our associated information was not provided. As mentioned above, these studies convey why this device can treat several types of neuropathies.

Concerning side effects, it should be discussed whether the heat effect generated by radiofrequency would damage nerves. Based on Sluijter et al.‘s study, if the temperature is above 45 °C, it will cause irreversible nerve damage, which is the harm that may be done by traditional radiofrequency [29]. Fortunately, the device we applied to our patient adopted pulsed radiofrequency, which never exceeded 42 °C. This advantage is achieved by using high-voltage bursts (45 V) in a short period (20 msec), followed by a silent phase (480 msec) for heat elimination, which keeps the target tissue below 42 °C [30]. As for our patient, we used infrared thermal imager to measure the temperature at the treatment site, and it showed 35 °C–36 °C which was only higher 1 °C–2 °C than the other side and was never above 42 °C during every treatment session.

One case is the limitation of our study because we only have encountered one patient with foot drop in our rehabilitation outpatient clinic so far. We hope that in the future, if we can find more patients with foot drop, we are also willing to use STIMPOD NMS460 for their treatment and record the treatment data, any side effect and degree of patients’ satisfaction, which can increase sample size and make results more convincing.

## 3 Conclusion

Foot drop markedly confines everyday activities of persons suffering from it. Therefore, there is a need for an optimized strategy for its treatment. With a noninvasive manner, STIMPOD NMS460, our patient got a huge improvement on her foot drop in three months and had no side effects. Owing to the benefits on nerve injury STIMPOD NMS460 may possess based on the aforementioned literature and the treatment effect on our patient, we believe that this device is fairly promising and may be qualified to be used in other patients with foot drop.

## Ethics approval and consent to participate

The study was approved by VGHKS institutional review board (KSVGH20-CT7-16). Also, we obtained the written consent allowing us to record the treatment process and to write it up as a case report from the patient.

## Consent for publication

The patient has given written consent for her data and photos for publication.

## Duplicate publication

The article we submitted has not been published previously, and it is not under consideration for publication elsewhere, in full or in part.

## Funding

This research received no specific grant from any funding agency in the public, commercial, or not-for-profit sectors.

## CRediT authorship contribution statement

**Yu-Cheng Chang:** Writing – review & editing, Writing – original draft, Project administration, Formal analysis, Data curation, Conceptualization. **Yuan-Ping Chao:** Data curation, Conceptualization. **Shin-Tsu Chang:** Writing – review & editing, Supervision, Resources, Methodology, Formal analysis, Data curation, Conceptualization.

## Declaration of competing interest

The Authors declare that there is no conflict of interest.

## Figures and Tables

**figs1:**